# Unusual Glycosaminoglycans from a Deep Sea Hydrothermal Bacterium Improve Fibrillar Collagen Structuring and Fibroblast Activities in Engineered Connective Tissues

**DOI:** 10.3390/md11041351

**Published:** 2013-04-23

**Authors:** Karim Senni, Farida Gueniche, Sylvie Changotade, Dominique Septier, Corinne Sinquin, Jacqueline Ratiskol, Didier Lutomski, Gaston Godeau, Jean Guezennec, Sylvia Colliec-Jouault

**Affiliations:** 1 Biochemistry Department, Dental School, Paris Descartes University, PRES Sorbonne Paris Cité, 1 rue Maurice Arnoux, Montrouge 92120, France; E-Mails: gueniche_farida@yahoo.fr (F.G.); godeau_g@yahoo.fr (G.G.); 2 UMR CNRS 7244, CSPBAT-LBPS, UFR SMBH, Paris 13 University, PRES Sorbonne Paris Cité, 74 Rue Marcel Cachin, Bobigny 93017, France; E-Mails: changotade@univ-paris13.fr (S.C.); lutomski@smbh.univ-paris13.fr (D.L.); 3 Laboratory of Biotechnology and Marine Molecules, IFREMER, B.P. 21105 44311 Nantes, France; E-Mails: corinne.sinquin@ifremer.fr (C.S.); jacqueline.ratiskol@ifremer.fr (J.R.); guezennec.jean@wanadoo.fr (J.G.)

**Keywords:** marine hydrothermal bacteria, glycosaminoglycan-mimetic, collagen, matrix metalloproteinases, dermal fibroblast, polysaccharides, tissue engineering

## Abstract

Biopolymers produced by marine organisms can offer useful tools for regenerative medicine. Particularly, HE800 exopolysaccharide (HE800 EPS) secreted by a deep-sea hydrothermal bacterium displays an interesting glycosaminoglycan-like feature resembling hyaluronan. Previous studies demonstrated its effectiveness to enhance *in vivo* bone regeneration and to support osteoblastic cell metabolism in culture. Thus, in order to assess the usefulness of this high-molecular weight polymer in tissue engineering and tissue repair, *in vitro* reconstructed connective tissues containing HE800 EPS were performed. We showed that this polysaccharide promotes both collagen structuring and extracellular matrix settle by dermal fibroblasts. Furthermore, from the native HE800 EPS, a low-molecular weight sulfated derivative (HE800 DROS) displaying chemical analogy with heparan-sulfate, was designed. Thus, it was demonstrated that HE800 DROS mimics some properties of heparan-sulfate, such as promotion of fibroblast proliferation and inhibition of matrix metalloproteinase (MMP) secretion. Therefore, we suggest that the HE800EPS family can be considered as an innovative biotechnological source of glycosaminoglycan-like compounds useful to design biomaterials and drugs for tissue engineering and repair.

## 1. Introduction

Traumatic lesions, chronic degenerative diseases and fibroses affect a variety of organs and tissues, which are characterized by alterations of the dialogue between cells and their microenvironment. Indeed, tissue homeostasis is under the strict control of cells, through the secretions of growth factors and cytokines or through the synthesis and degradations of extracellular matrix macromolecules. In turn, any changes in the extracellular matrix structure or composition also affect the cell behavior. 

In order to promote wound healing or replace large tissue loss, the use of matrix macromolecules as drugs or biomaterials can be a clever approach. Among extracellular matrix components, glycosaminoglycans (GAGs) are of particular interest, because these linear polyanionic polysaccharides display a very weak immunogenicity and play a major role in tissue remodeling [1]. GAGs are ubiquitously found in all animal tissues and can be located into the extracellular matrix, on the cell surface or in the intracellular compartment. These polymers are composed of disaccharide repeating units containing both uronic acid (or neutral sugar) and amino sugar. These GAGs can be sulfated (chondroitin-sulfates, dermatan-sulfates, heparin/heparan-sulfates and keratan-sulfates) or not (hyaluronic acid). Sulfated glycosaminoglycans are covalently bound to a protein-core to form proteoglycans, while hyaluronic acid (HA) does not [2]. Furthermore, sulfated GAGs display molecular weights varying between ten and one hundred kilodaltons, whereas HA has a very high molecular weight (5000 to 7000 kDa) [3]. GAGs interact with a wide range of proteins involved in cell metabolism and tissue remodeling. Thus, these polysaccharides display many biological effects on all stages of tissue repair, as well as on regulation of inflammatory response [1,2]. For example, cell surface heparan-sulfates are required to transmit extracellular signal [4,5,6] and to regulate bioavailability [7,8] of heparin-binding growth factors, such as fibroblast growth factors (FGFs), heparin-binding endothelial growth factor (HB-EGF) or vascular EGF (VEGF). Their ability to give structure to matrix macromolecules, such as collagens, matrix glycoproteins and elastic fibers, was also described [9,10,11,12,13]. 

Other studies demonstrated that both heparin and chondroitin-sulfate directly inhibit serine-proteinase activities [14,15] and modulate matrix metalloproteinase syntheses in cell culture [16,17]. Furthermore, heparan-sulfates of endothelial cell surfaces are key players in leukocyte homing and rolling and show good affinities for chemokines and selectins [18,19].

Hyaluronic acid (HA), is a major player in tissue remodeling. The hallmark of this polysaccharide is its ability to form hydrogels performing rheological properties of synovial fluids and joint lubrication and creating space for cell migrations during wound healing and embryogenesis [3]. During skin wound healing, HA could prevent fibrosis [20]. For example, high amounts of HA are produced during fetal skin repair, which is characterized by scarless wound healing [3], whereas synthesis of this GAG is dramatically downregulated during keloid scar formations [21]. Furthermore, HA oligosaccharides stimulate endothelial cell proliferation, migration and differentiation trough activation of specific cell receptors (CD44 and RHAMM) [22]. These HA oligosaccharides are also involved in inflammatory response after injury by stimulation of macrophage cytokine secretions through a CD44 signaling pathway [19]. 

Therefore, these polysaccharides could be advantageously used in tissue therapy and could be proposed as pharmacological agents or biomaterials for tissue repair and tissue engineering. Unfortunately, the strong anti-coagulant activity displaying by some of them (heparin, heparan-sulfate), their animal origin associated with potential adventitious agents (e.g., viruses, prions) and unreliable availability (cost, volume) restrict their use in human.

Nowadays, marine microorganisms can be regarded as innovative biotechnological alternatives to produce bioactive polysaccharides mimicking glycosaminoglycan properties [1,23]. Indeed, the sea harbors flourishing life forms and, particularly, organisms with outstanding metabolisms. Thus, in the deepest abysses, despite extreme conditions, hydrothermal vent chimneys support complex ecosystems comprising eubacteria, archaebacteria, protozoan’s, arthropods, mollusks and fish. Among the deep sea hydrothermal microorganisms, a wide variety of mesophilic bacteria had been isolated to produce innovative compounds of biopolymers, such as polyesters (polyhydroxyalkanoates) and polysaccharides (exopolysaccharides) [24,25].

The latter ones are mainly produced by *Alteromonas*, *Pseudoalteromonas* or *Vibrio* species and are secreted as high-molecular weight heteropolysaccharides (>10^6^ Da). Marine EPS are usually composed of branched repeating units from four to 10 sugars mainly composed of neutral sugars and uronic acids, which bear substituents, such as sulfate, phosphate, pyruvate or lactate [26].

**Figure 1:**
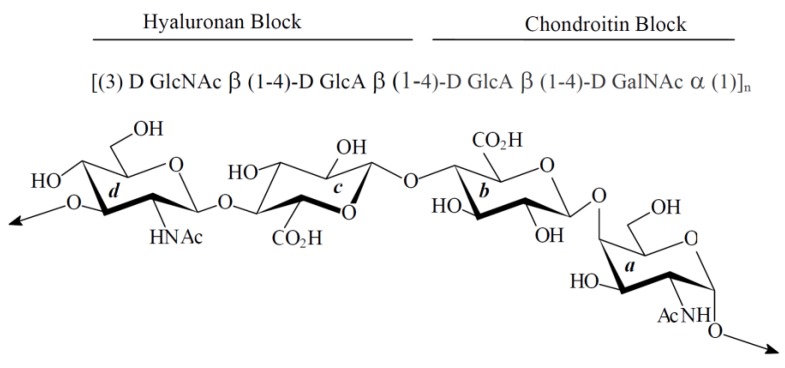
HE800 exopolysaccharide (HE800 EPS) is a glycosaminoglycan: Tetrasaccharidic unit of HE800 EPS, as described by Rougeaux *et al*. (1999) [28]. The HE800 EPS repeating unit comprises the specific hyaluronan disaccharide linked to the specific non-sulfated chondroitin disaccharide.

However, one of them, secreted by the *Vibrio diabolicus* bacterium HE800 strain (CNCM number: I-1629), is very peculiar. Indeed, HE800 EPS (Hyalurift^®^ trade mark) displays a glycosaminoglycan-like structure, rarely described for a bacterial exopolysaccharides [27,28]. Native high-molecular weight HE800 EPS is constituted by a linear non-sulfated tetrasaccharidic repeating unit, including equal amounts of glucuronic acid and hexosamine (*N*-acetyl-glucosamine and *N*-acetyl-galactosamine), which can be regarded as the combination of both hyaluronan and non-sulfated chondroitin disaccharidic repeating unit (Figure 1) [27,28]. Thus, due to both high uronic acid and hexosamine contents, this EPS exhibits chelating activity against heavy metals [29]. More interestingly, the ability of native high-molecular weight form of HE800 EPS to enhance *in vivo* bone repair has been demonstrated in a rat model [30].

So, we assumed that native HE800 EPS could be a good candidate as biomaterial to design biocompatible scaffolds and, especially, in association with fibrillar collagens. After sirius red staining, we studied the structure of mixed films containing both fibrillar acido-soluble collagen I and native HE800 EPS. Reconstructed dermis containing collagen I and native HE800 EPS were also performed in order to study collagen structuring and tissue settling by dermal fibroblasts. Thus, the structure and ultrastructure of these composite scaffolds were studied by conventional histology and electron microscopy, and after image analyses, we calculated changes in the tissue cell densities during culture. 

Additionally, taking advantage of its glycosaminoglycan-like features, we designed a low-molecular weight, deacetylated and oversulfated HE800 EPS derivative (HE800 DROS) with the purpose of mimicking the properties of heparan-sulfates. HE800 DROS was then assayed on human dermal fibroblast cultures to observe their potential effects on cell proliferation and matrix metalloproteinase secretions.

## 2. Results

### 2.1. Characterization of Native HE800 EPS and Its Sulfated Low-Molecular Weight Derivative

**Figure 2:**
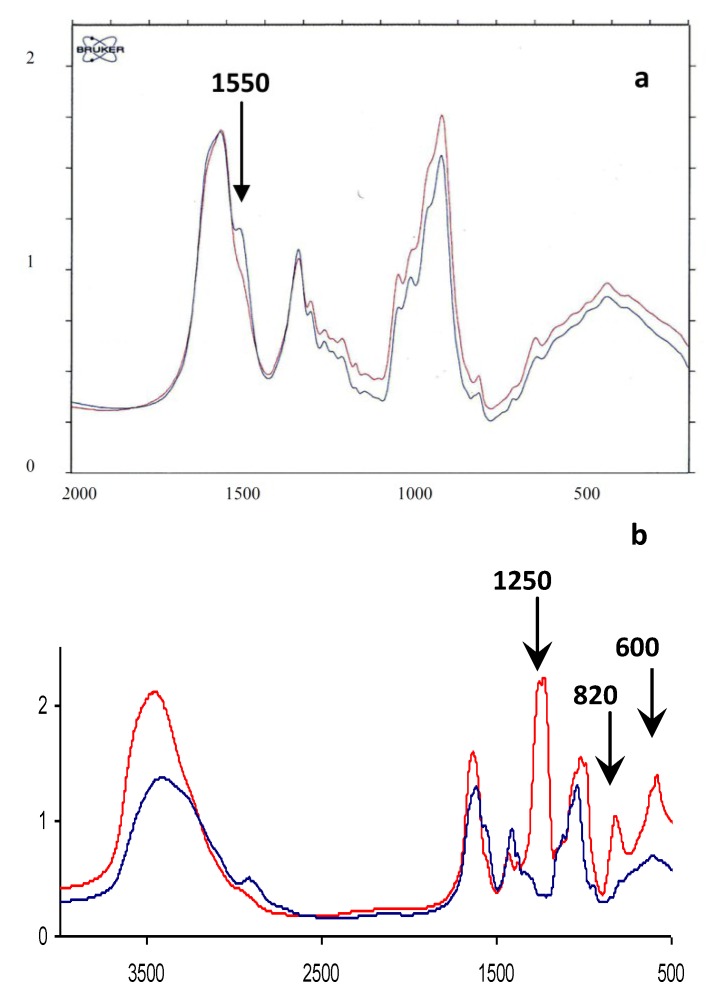
FTIR spectra of HE800 EPS and its derivatives. (**a**) Infrared spectrum of depolymerized HE800 EPS before (blue line) and after (red line) deacetylation; (**b**) Infrared spectrum of depolymerized HE800 EPS (blue line) and HE800 DROS (red line).

As presented in Table 1, the native HE800 EPS displays a high-molecular weight (850,000 g/mol), and its chemical composition is in accordance with its described structural feature containing 38 and 39% (w/w) of hexosamines and uronic acids, respectively. On the other hand, the sulfated low-molecular weight derivative shows a molecular weight of 22 kDa with a low polydispersity (Ip = 1.4). Because of the high rate of sulfate (34%, w/w), the content of both hexosamine and uronic acid is only 20% (w/w), but is still in agreement with the initial structure of the HE800 EPS. Figure 2 shows the infrared spectra of the EPS before and after deacetylation and sulfation steps. The deacetylation reaction is marked by the decrease in the shoulder observed at 1550 cm^−1^ in the blue curve of Figure 2a, which corresponds to the decrease in *N*-acetyl groups and suggests an increase of N–H of secondary amide groups. After sulfation of deacetylated and depolymerized HE800 (Figure 2b, blue curve) ester sulfate bands are observed at 1250, 820 and 600 cm^−1^.

**Table 1:** Chemical compositions and molecular weights of native HE800 EPS and oversulfated HE800 EPS derivative (HE800 DROS).

	Native HE800 EPS	HE800 DROS
Neutral sugars ^a^ (%)	0	0
Hexosamines^ a^ (%)	38	20
Uronic acids ^a^ (%)	39	20
SO_3_Na ^b^ (%)	0	34
Mw^ c^ (g/mol)	850,000	22,000
Ip	Not determined	1.4

^a ^colorimetric analysis; ^b ^calculated from sulfate content determined with elemental analysis; ^c ^determined by high pressure size exclusion chromatography (HPSEC) using pullulans as standards.

### 2.2. Effect of HE800 EPS on Collagen Film Structuring

**Figure 3:**
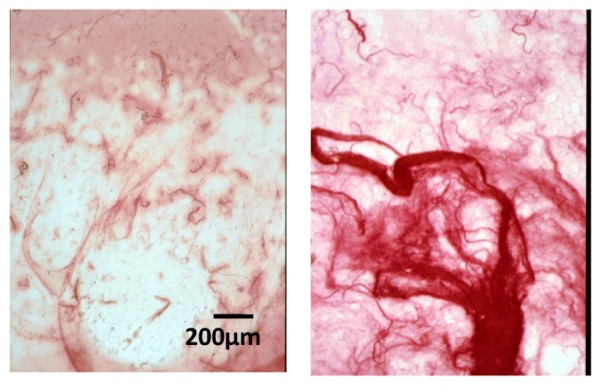
HE800 EPS promotes collagen aggregation on films: Films were stained with sirius red and observed under transmitted light (magnification ×26). (**a**) Collagen alone (40 μg); and (**b**) collagen (40 μg) + HE800 EPS (200 μg); scale bar = 200 μm.

Films were performed after evaporation of solutions containing 40 μg of collagen I and different amounts of native HE800 EPS. The first type of films (films 1) are composed of collagen alone (40 μg) (Figure 3a), and the second type (films 2) are composed of collagen (40 μg) and HE800 EPS (200 μg) (Figure 3b). After sirius red staining under transmitted light, the collagen network appears in film 2 as a dense network composed of long fine strands, whereas film 1 contains virtually none. Furthermore, we noticed that some short fibers were observed when equivalent quantities of collagen and HE800 EPS were used (not shown). Finally, all films are effectively stained with sirius red, but under polarized light, only a birefringent fibrillar network is observed in the case of film 2 (data not shown).

### 2.3. Effect of Native HE800 EPS on Collagen Matrix Structuring in Reconstructed Connective Tissue (RCT)

**Figure 4:**
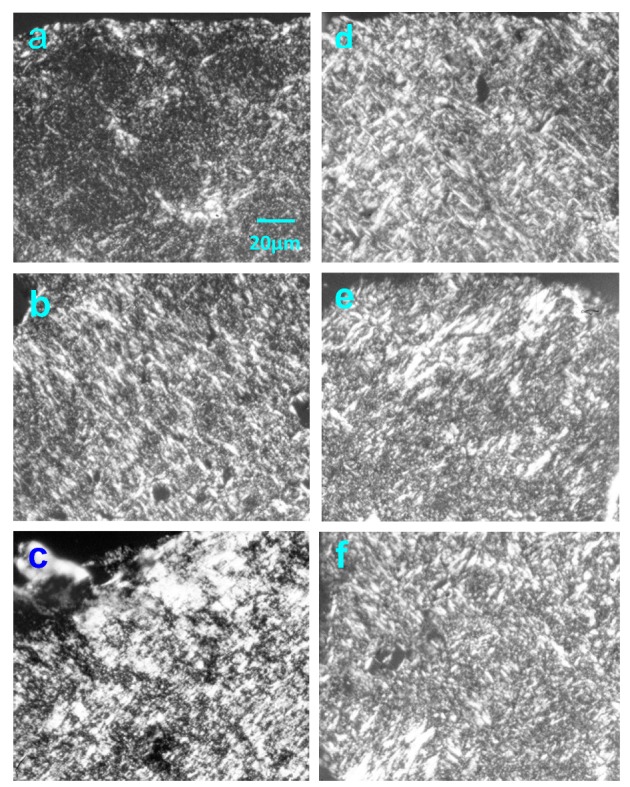
HE800 EPS enhances reconstructed connective tissue structuring: Paraffin embedded reconstructed connective tissue (RCT) sections. After Sirius red staining, sections were observed under polarized light microscopy: (**a**) and (**d**) RCT controls; (**b**) and (**e**) RCT containing 150 μg of HE800 EPS; (**c**) and (**f**) RCT containing 75 μg of HE800 EPS. (**a**), (**b**) and (**c**) 11 days-RCT; (**d**), (**e**) and (**f**) 40 days-RCT. Magnification ×160; scale bar = 20 μm for all.

Native HE800 EPS was used to perform composite dermal matrices containing living human dermal fibroblasts. Thus, reconstructed connective tissues (RCTs) are composed of collagen I alone (controls) or collagen I and HE800 EPS in various proportions: 20%, 10%, and 5% of EPS relative to the total amount of collagen (3 mg), which corresponds to 300 μg, 150 μg and 75 μg of HE800 EPS per RCT, respectively. Figure 4 presents sirius red stained paraffin-embedded sections of RCTs fixed after 11 and 40 days of culture. Under polarized light, 11-day RCT controls (Figure 4a) display an extracellular matrix presenting some thin and sparse collagen bundles, whereas 11-day RCTs containing 150 μg of HE800 EPS (Figure 4b) present a more structured extracellular matrix, containing some thicker collagen bundles. A dense extracellular matrix with numerous thick collagen bundles is observed, after 11-day RCTs containing 75 μg of HE800 EPS (Figure 4c). Forty-day RCT controls present numerous collagen bundles and matrix architecture similar to the 11-day RCTs containing 150 μg of HE800 EPS. Both 40-day RCTs containing 150 μg and 75 μg of HE800 EPS present a very dense numerous thick collagen bundles.

### 2.4. Effect of Native HE800 EPS on Collagen Fibrillogenesis in Reconstructed Connective Tissue

**Figure 5:**
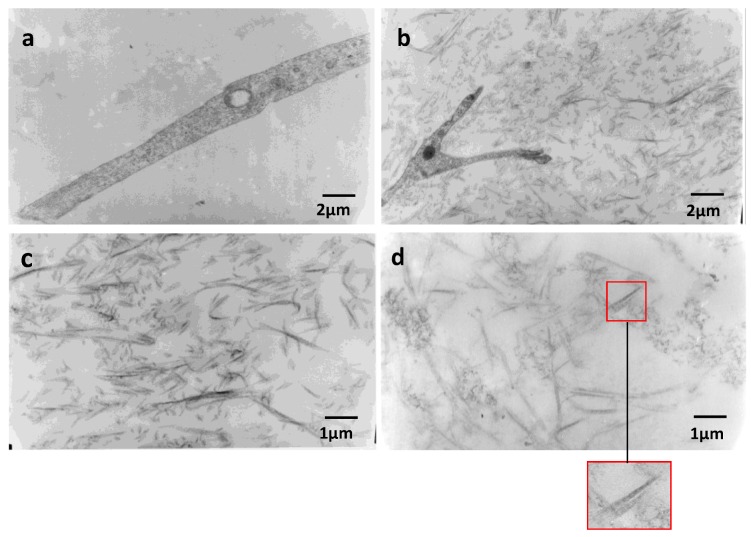
HE800 EPS promotes collagen fibrillogenesis in reconstructed dermis: 11-day RCT sections were observed by transmission electron microscopy. (**a**) Cell filopodia observed in RCT controls; (**b**) cell filopodia observed in RCT containing 150 μg of HE800 EPS. (**c**) Collagen fibrils in RCT containing 75 μg of HE800 EPS; (**d**) collagen fibrils in RCT containing 150 μg of HE800 EPS; the insert highlights periodic striation of collagen fibers. (**a**) and (**b**) magnification ×10,000; (**c**) and (**d**) magnification ×20,000.

Electron microscopy was carried out on equivalent connective tissues cultured for 11 days. In all RCTs, cells have a good ultrastructural state. Sparse collagen fibers can be observed in RCT controls (magnification ×10,000) (Figure 5a), whereas in RCTs containing 150 μg of HE800 EPS, numerous fibrillar elements included in a gel that could be formed by the exopolysaccharide are observed (Figure 5b). At a magnification of ×20,000, as observed in Figure 5c,d, the RCTs containing 75 μg or 150 μg of HE800 EPS, respectively, display numerous collagen fibers throughout their extracellular matrix. These fibers are marked by a characteristic 67 nm periodic striation, as shown in the insert on Figure 5d. 

### 2.5. Effect of Native HE800 EPS on Fibroblast Densities in Reconstructed Connective Tissues

Tissue contraction obtained by the daily measure of lattice diameter is similar in all RCTs. After the eleventh day, tissue contractions are almost complete. The number of cells settled in each RCT varies, depending on the culture time, between 120,000 and 180,000 cells; volumetric cell densities in the extracellular matrix fluctuate between 2700 and 4700 cells/mm^3^. The number of RCT peripheral cells represents 2% to 12% of the total number of cells. Because of the small volume of this area, the peripheral cell densities appear much larger than those settled within extracellular matrix (100,000 to 250,000 cells/mm^3^).

Variations of the cell densities in the extracellular matrix and at the periphery of RCTs between 11 and 40 days of culture are shown in Table 2. Cell densities in the extracellular matrix of RCT controls do not vary between 11 and 40 days of culture (Table 2). However, in the RCTs containing HE800 EPS, about a 50% to 70% increase is observed. No significant decrease in the peripheral cell density of the RCT controls is observed, but the peripheral cell densities of RCTs containing HE800 EPS are significantly reduced.

**Table 2:** HE800 EPS favors dermal fibroblast migrations and proliferations in reconstructed connective tissues: RCT sections were stained by haemalun-eosin reagents to distinguish cell nuclei to extracellular matrix. Cells were counted, and cell densities were calculated as cell numbers per RCT volume unit (see 4.5 in the Experimental Section). Cell density variations in each RCT area were compared between the eleventh and fortieth day. ** *p* ≤ 0.01 and *** *p* ≤ 0.001.

	Extracellular Matrix	Periphery
Control	−2.7%	−22.9%
HE800 EPS (300 μg)	+48.9% (**)	−53.6% (**)
HE800 EPS (150 μg)	+68.1 (**)	−68.4% (***)
HE800 EPS (75 μg)	+54.5% (**)	−75.2% (**)

### 2.6. Effect of HE800 DROS Derivative on Matrix Metalloproteinase Secretions

**Figure 6:**
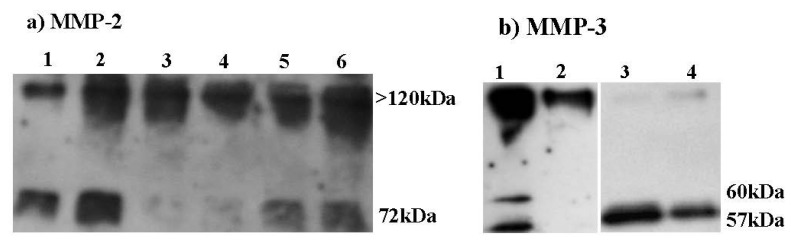
HE800 DROS inhibits matrix metalloproteinase (MMP) secretions (Western blots): (**a**) MMP-2: lane 1: control; lane 2: IL-1β (100 U/mL); lane 3: HE800 DROS (10 μg/mL); lane 4: HE800 DROS (100 μg/mL); lane 5: HE800 DROS (10 μg/mL), +IL-1β (100 U/mL); lane 6: HE800 DROS (100 μg/mL), +IL-1β (100 U/mL); (**b**) MMP-3: lane 1: control; lane 2: HE800 DROS (10 μg/mL); lane 3: IL-1β (100 U/mL); lane 4: HE800 DROS (10 μg/mL), +IL-1β (100 U/mL).

Figure 6a shows the Western blot of gelatinase A (MMP-2) secreted by confluent dermal fibroblasts, which were incubated for 48 h in serum-free media containing or not IL-1β (100 U/mL), with or without HE800 DROS (10 or 100 μg/mL). In all culture conditions, the free form of proMMP-2 (72 kDa) and a high molecular weight complex form of MMP-2, probably associated to a tissue inhibitors of matrix metalloproteinases (>120 kDa), are observed. IL-1β alone (100 U/mL) increases the gelatinase A basal level (Figure 6a). HE800 DROS (10 or 100 μg/mL) strongly decreases the secretion of free proMMP-2, but not MMP-2 complexes.

The same procedure was performed to study stromelysin-1 (MMP-3) secretion (Figure 6b). MMP-3 is present as both free pro-isoforms (57 and 60 kDa) and high molecular weight complexes with a tissue inhibitor of metalloproteinase (TIMP) (>100 kDa). Without IL-1^®^, HE800 DROS decreases secretions of both free and complex isoforms of MMP-3. The addition of IL-1^®^ in culture media strongly increases the secretion of 57 and 60 kDa proMMP-3 isoforms. With IL-1^®^, secretion of proMMP-3 is inhibited by HE800 DROS, but a slight increase in MMP-3 high molecular weight complexes can be noticed.

### 2.7. Effect of HE800 DROS on Dermal Fibroblast Proliferation

**Figure 7:**
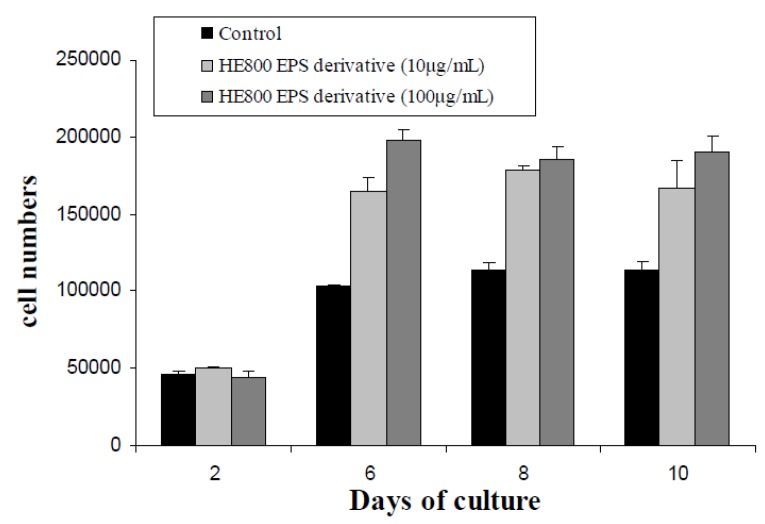
HE800 DROS enhances proliferation of human dermal fibroblast: cells were incubated with HE800 EPS DROS (10 and 100 μg/mL) during 10 days. Cells were counted after two, four, six and 10 days of culture.

Figure 7 presents the HE800 DROS effect on dermal fibroblast proliferation in two dimensional cultures. The HE800 DROS stimulates fibroblast proliferation during the exponential proliferative phase between day 2 and day 6. At day 6, large increases of proliferation were noticed (60% and 90% with 10 and 100 μg/mL of HE800 DROS, respectively). After day 6, cell proliferation curves reach a stationary phase. 

## 3. Discussion

Initially, the ability of a native high-molecular weight form of the EPS produced by *Vibrio diabolicus* (HE800 EPS) to constitute a biocompatible scaffold was tested. Using the capability of acido-soluble atelocollagen I to auto-associate into fibrils after pH neutralizing [31], reconstructed extracellular matrices containing human fibroblasts and various amounts of HE800 EPS were performed. Thus, using conventional histology and electron microscopy, it has been shown that the addition of HE800 EPS, during collagenous matrix remodeling, increases the formation of *D*-periodic striated collagen fibrils (*D* = 67 nm) and promotes human dermal fibroblasts migration and proliferation.

As reported by Stuart and Spanich (2008) [32], it is difficult to conclude about general mechanisms leading to the effects of glycosaminoglycans on collagen fibrillogenesis, because the studies regarding the interactions between glycosaminoglycans and collagens are carried out on reconstructed tissue models that differs in pH, ionic concentration or macromolecular ratios, *etc.*

However, it can be inferred that like chondroitin, dermatan sulfate, heparin or hyaluronan [9,32,33], both the linear structure and anionic feature of HE800 EPS allow interactions with rod-like cationic polymers that are fibrillar collagens. Therefore, HE800 EPS might favor the deposit of tropocollagen molecules forming fibrils before enzymatic crosslinking.

The proliferation and migration of fibroblasts across different regions of reconstructed connective tissues during the culture were also studied. Previous work suggested that fibroblastic cells within the reconstructed tissue models preferentially proliferate and migrate in a two-dimensional environment after digestion of surrounding extracellular matrix (Helary *et al*. 2005) [34]. We also observed that proliferating cells are largely located on the RCT surface [35].

In order to assess the number of both fibroblasts that settled in the extracellular matrix and fibroblasts adhering and proliferating on the surface of the reconstructed tissues, stained histological sections were studied by image analysis. Between the eleventh and the fortieth day, cells located on the RCT surface massively migrate into the extracellular matrix of RCTs containing HE800 EPS. This phenomenon explains the about 50% increase of fibroblasts densities in the extracellular matrix of the RCTs containing the polysaccharide. 

It was previously observed that high-molecular weight hyaluronic acid promotes fibroblast migration in both fibrin and collagen lattices [36,37,38]. These effects may be caused by the modification of matrix macromolecule architectures, which involves a cytoskeleton rearrangement suitable for fibroblast migrations [36,37]. We can thus hypothesize that hyaluronan, as well as HE800 EPS are able to modify the collagen fibril layout, revealing specific cell adhesion sites on collagen fibrils and creating spaces between collagen bundles, which both facilitate fibroblast migrations across the extracellular matrix. Therefore, promotion of both collagenous matrix making and cell migration may explain the HE800 EPS capability to improve *in vivo* bone repair.

The addition of the HE800 EPS forming gel in critical size defects performed in rat calvaria induces after 15 days an almost complete bone healing, whereas control animals (without treatment) or animals treated with a simple collagen sponge do not demonstrate any significant healing. This healing is furthermore characterized, in the presence of HE800 EPS, by a total restoration of the cortical and trabecular bone structures and neoangiogenesis along the well-organized trabecular bone [30].

Thus, during tissue repair, HE800 EPS act as a biocompatible exogenous provisional matrix, favoring extracellular matrix macromolecule deposits and migrations of both osteoblastic and endothelial cells. These data and our results allow us to suppose that native HE800 EPS could be a useful bio-reactive scaffold in skin or cartilage engineering and repair. 

Furthermore, we were interested in the peculiar structure of the HE800 EPS, which can be regarded as a precursor molecule in order to design chemical derivatives close to sulfated glycosaminoglycans (GAGs), such as heparan sulfate or chondroitin sulfate. For this purpose, native HE800 EPS is first depolymerized by a free-radical reaction and then chemically deacetylated and sulfated. 

HE800 DROS was demonstrated as a potent inhibitor of the complement cascade with the advantage of a ten-times less anticoagulant activity than heparin [35]. Furthermore, in this study, HE800 DROS promotion of dermal fibroblast proliferation during the first days of culture corresponding to exponential cell growth phase has been shown. Thus, HE800 DROS may interact with heparin-binding growth factors and promote their activities, as well as heparan sulfate [4].

We have also observed that the HE800 DROS derivative inhibits basal free proMMP-2 (72 kDa) secretions and IL-1^®^ mediated stimulation of both free MMP-2 and MMP-3 secretions. It has been reported that both heparin and chondroitin sulfate inhibit the overexpression of MMP-1 and MMP-3 by IL-1^®^ in mesenchymal cells, such as mesangial cells [39], gingival fibroblasts [17], synovial fibroblasts or chondrocytes [40,41]. Thus, mechanisms explaining inhibition of IL-1^®^ signaling by sulfated GAGs may involve the inhibition of protein kinase C (PKC)-dependent pathways [16] or alteration in intracellular trafficking mediated by calcium [42]. We can also suppose that the decrease of free MMPs is caused by an increased level of high-molecular weight complex formation between MMPs and TIMPs, as observed with heparin and heparan-mimetics [43,44,45].

These data suggest that in spite of the lack of l-iduronic acid, the properties of HE800 DROS derivative are very close to that of heparan-sulfate. Indeed, it is well accepted that the richness of l-iduronic acid increases heparan-sulfate chain flexibility, which improves its affinity to specific ligands, like FGFs [46,47,48]. However, unlike classical GAGs, the HE800 EPS is characterized by a direct osidic bound between the two glucuronic acids of its tetrasaccharidic repeating unit (Figure 1) [28]. We can suppose that this peculiar structural feature favors HE800 EPS interactions with heparan-sulfate-specific ligands.

## 4. Experimental Section

### 4.1. HE800 EPS

HE800 EPS is naturally produced under controlled conditions by fermentation of a non-pathogenic marine bacteria, *Vibrio diabolicus*, HE800 strain (CNCM: I-1629). This strain was isolated from a worm in a deep sea hydrothermal field in the East Pacific rose by IFREMER during the French-American cruise, HERO, in October 1991. HE800 EPS is obtained from fermentation broth by centrifugation and ultrafiltration steps, according to Raguenes *et al*. (1997) [27]. 

HE800 DROS was prepared as previously described in U.S. Patent 2008/0,131,472 [35]. The process briefly consists in a free radical depolymerization step in the presence of H_2_O_2_ to obtain a low molecular weight EPS (HE800 DR). The depolymerized HE800 was then deacetylated with NaOH and NaBH_4_ and sulfated with pyridine sulfur trioxide performed in DMF on the pyridinium salt of HE800 DR.

The native EPS and its derivatives were then characterized. For the determination of sugar composition, monosaccharides were determined by gas chromatography (GC) analysis of trimethylsilyl derivatives after acidic methanolysis [49,50]. Total carbohydrate was estimated by the modified phenol-sulfuric acid method of Dubois *et al.* (1956) [51], using glucose as standard. Uronic acids were determined by the m-hydroxydiphenyl of method from Blumenkrantz *et al.* (1973) [52], using glucuronic acid as the standard. Protein content was estimated according to the method, Bicinchoninic Acid (BCA) Protein Assay [53]. Nitrogen, hydrogen, carbon and sulfur contents were determined by elemental analysis by the Central Microanalysis Department of the CNRS (Gif-sur-Yvette, France). Sulfate content (sodium salt) was deduced from sulfur analysis according to the following relation: sulfate group% = 3.22 × S%. The molecular weight of the polysaccharide was determined by size exclusion chromatography using a PL aquagel-0H, (Varian) column, a pump and an injector (Kontron Instrument, Montigny le Bretonneux, France). Elution was performed at 1 mL·min^−1^ with 0.1 M ammonium acetate. Samples were prepared at 2 mg/mL, filtrated on a 0.45 μm membrane prior to injection (100 μL). The detection was performed using an Erma refractive index (RI) detector. A calibration curve was done with pullulan standards (Supelco, Bellefonte, PA, USA), and the data were computed using Aramis software (Varian, Les Ullis, France) for Mw (weight-average molecular mass), Mn (number-average molecular mass) and I (polydispersity) calculation. Fourier transform infrared analysis was performed on pellets obtained by careful grinding of a mixture of 2 mg of EPS with 200 mg of potassium bromide (KBr), FTIR grade. Infrared spectra were recorded on a BOMEM M100 Fourier Transform Infrared Spectrometer with a resolution of 4 cm^−1^.

### 4.2. Human Dermal Fibroblasts

Cells were obtained from prepuces of healthy subjects aged 3–4. Patients’ parents gave their informed consent for biopsies, according to the Helsinki declaration. Primary and secondary cultures were obtained, as previously described [45]. Briefly, skin biopsies were cut into small pieces, and adherent explants were cultured in appropriate media (DMEM/Fetal Calf Serum (FCS) 20%/penicillin (100 IU/mL)/streptomycin (100 μg/mL)/amphotericin B (2 μg/mL)) in a humidified atmosphere of 5% CO_2 _at 37 °C. After three weeks, explants were thrown out, and cells were trypsinized and seeded onto new culture flasks in complete media (DMEM/FCS 10%/streptomycin (100 μg/mL)/penicillin (100 IU/mL)). Then, dermal fibroblasts were sub-cultured weekly until the assays. 

### 4.3. Collagen and Collagen-HE800 EPS Films

The collagen used was an acid-soluble collagen I (2 mg/mL) obtained from rat tail tendon (Insitut Jacques Boy, Reims, France). Collagen films were carried out by depositing 200 μL of collagen solution (40 μg in total) in wells of chambered cover glass (2 cm^2^) (Labtek™, Thermo Fischer-Scientific, Courtaboeuf, France). Composite-collagen films were performed by depositing a mixture of 200 μL of collagen (40 μg in total) and native HE800 EPS (5, 40 or 200 μg in total) at the surface of wells (2 cm^2^). Then, wells were placed under a culture hood at 37 °C. After evaporation, films were formed and could be fixed by absolute ethanol at −20 °C and then rehydrated to be stained by sirius red F3ba [54]. Under polarized light, fibrillar collagens are birefringent and yet appear as yellow, red, green or white rods.

### 4.4. Reconstructed Connective Tissues (RCT)

RCT were prepared in 60-mm-diameter bacteriologic Petri dishes, as previously described [55], by mixing 2.75 mL of concentrated Dulbecco EMEM with FCS, 1.5 mL of type I collagen solution (2 mg/mL in acetic acid (0.1%) from rat tail tendons) (Institut Jacques Boy, Reims, France), 0.25 mL NaOH (0.1 M) and 0.5 mL of dermal fibroblast suspension (3 × 10^5^ cell/mL). A collagen gel was obtained rapidly when dishes were placed at 37 °C in a CO_2_ incubator. After 1 h, a slight shaking was performed in order to detach RCTs from the edges. Due to the active organization of collagen fibrils by fibroblasts, the collagen matrix gradually contracts over a few days. After 14 days, the contraction phase is finished, and the RCT is considered stabilized. To design composite RCT, HE800 EPS was added to the collagen before cell additions, at the rate of 150 μg, 300 μg or 600 μg per lattice (respectively, 5%, 10% and 20% of the total amount of the collagen). Culture media were changed every week. 

### 4.5. RCT Histology and Cell Counting by Image Analysis

RCTs were fixed for 24 h in a phosphate buffered 4% paraformaldehyde solution, then dehydrated and embedded in paraffin using standard procedures. Serial 8 μm thick tissue sections were performed and prepared for histochemistry and image analyses. Fibrillar collagen bundles were observed after sirius red staining, and cell nuclei can be distinguished from the collagen matrix after haemalun eosine-staining.

Tissue cell densities expressed as cell numbers per mm^3^ were determined, as described previously (Miller *et al*. 2003) [55], using image analysis. Briefly, cells were counted after 11 and 40 days of culture in 30 different fields per RCT. Each field has a surface defined by microscope magnification and a thickness of 8 μm (thickness section). Two groups of cells can thus be differentiated according to their geographical situation: Firstly, the cells located inside the RCT (extracellular matrix), and secondly, the cells located at its surface (periphery).

In order to calculate cell densities, each RCT was considered as a cylinder, whose diameter (*D*) corresponds to the value measured just before inclusion in paraffin and whose height (*h*) corresponds to its thickness, as determined semi-automatically on histological sections with a Biocom station and the Imagenia 3000 program (Biocom, Les Ulis, France). The peripheral area was considered as a crown of 10 μm thickness, with height corresponding to the thickness of RCT (*h*). The cell number in each field was reported to the total volume of peripheral or extracellular matrix region to calculate the extracellular matrix or peripheral cell densities, which are expressed as cell numbers/mm^3^. 

### 4.6. Transmission Electron Microscopy

RCTs were fixed with glutaraldehyde (2%, w/v) in cacodylate buffer (0.15 M, pH 7.4) for one hour at 4 °C. After post-fixation in OsSO_4_ (2%) for 1 h and dehydration in a graded ethanol series at 4 °C, the samples were embedded in Epon 812 (Fluka). Semi-thin sections (1 μm thick) were obtained with a glass knife on a Richert Ultramicrotome and then stained with toluidine blue. After washing, the sections were dried and mounted in Eukitt. 50 nm thick ultra-thin sections were obtained with a diamond knife and then stained in uranyl acetate and lead citrate. Sections were examined in a JEOL JEM 100B electron microscope at 80 V. 

### 4.7. MMPs Studies in Bidimensional Fibroblast Cultures

Human dermal fibroblasts were grown in complete medium in 24-well plates until confluence. Complete media was then removed, and cells were rinsed twice with PBS. Cells were incubated for 48 h with serum-free media alone (control) or with serum-free media containing HE800 DROS (10 or 100 μg/mL) in the presence or not of IL-1^®^ (100 U/mL). 

### 4.8. Western Blot

Samples were mixed with non-reducing sample buffer (250 mM Tris, pH 6.8, 50% glycerol, 0.4% bromophenol blue and SDS 10%). As previously described in [44], gels were run under Laemmli conditions at 80 V for 15 min and then 160 V for 90 min. Proteins were transferred onto a polyvinylidene fluoride membrane (PVDF, from Merck Millipore, Molshem, France) at 80 V for 1 h. Non-specific binding sites were blocked for 1 h (nonfat milk, 5%, 0.05% Tween 20 in PBS) and washed in PBS (4 × 15 min). The membrane was incubated for 1 night at 4 °C with a monoclonal primary antibody directed against human MMPs (Calbiochem, WWR, Strasbourg, France, dilution 1/500 in PBS), washed, then incubated with an appropriate HRP secondary antibody (Sigma Aldrich, Saint Quentin Fallavier, France, dilution 1/750 in PBS). Peroxidase activity was detected by chemiluminescence.

### 4.9. Statistical Analysis

Variations of cell densities between day 11 and day 40 were compared using a non-parametric test (a Kruskal-Wallis test followed if it was significant by group comparison with the Mann-Whitney *U* test). Differences were considered significant if *p* ≤ 0.05.

## 5. Conclusion

**Figure 8:**
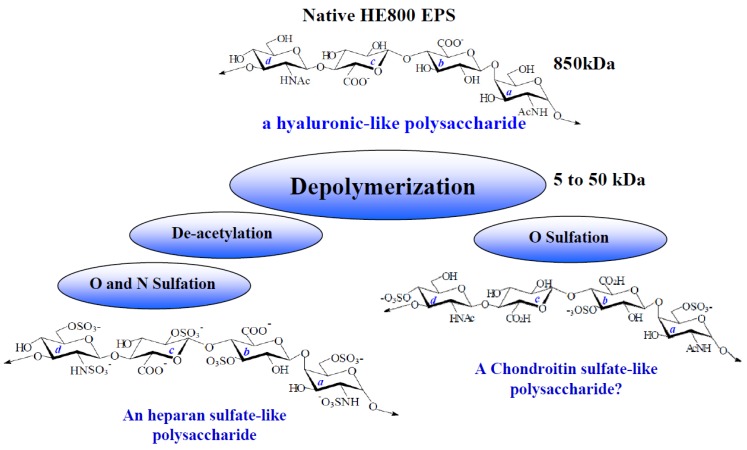
HE800 EPS and its derivatives: A new family of glycosaminoglycans.

In this work, we have shown that native form of HE800 EPS can be regarded as a new biomaterial to form scaffolds for tissue repair and engineering. Furthermore, due to its GAG-like structure, the HE800 EPS could also be regarded as a biotechnological precursor to design a new GAG family, replacing animal extracted glycosaminoglycans (Figure 8). Thus, marine biotechnology could allow the production of hyaluronan-like, chondroitin sulfate-like or heparan-sulfate-like polysaccharides, fulfilling both safety and availability requirements, which are essentials for health markets.
